# Correlates of Subjective Cognitive Decline in Black American Men

**DOI:** 10.14283/jpad.2024.162

**Published:** 2024-09-04

**Authors:** Darlingtina K. Esiaka, C. Nwakasi, A. Q. Briggs, D. F. Conserve, R. J. Thorpe

**Affiliations:** 1 Department of Behavioral Science, University of Kentucky College of Medicine, Lexington, KY USA; 2 Center for Health Equity Transformation, University of Kentucky College of Medicine, 760 Press Avenue, Suite 426, Rm 472, Lexington, KY 40536 USA; 3 Department of Human Development and Family Science, University of Connecticut, Mansfield, CT USA; 4 Department of Neurology, Alzheimer’s Disease Research Center, NYU Grossman School of Medicine, New York, NY USA; 5 Department of Prevention and Community Health, Milken Institute School of Public Health, George Washington University, Washington, DC, DC USA; 6 Program for Research on Men’s Health, Hopkins Center for Health Disparities Solutions, Johns Hopkins Bloobmerg School of Public Health, Baltimore, MD USA; 7 Johns Hopkins Alzheimer’s Disease Resource Center for Minority Aging Research, Johns Hopkins Bloobmerg School of Public Health, Baltimore, MD USA

**Keywords:** Sleep, neighborhood, cognitive decline, ADRD risk

## Abstract

**Background:**

Past research suggests that subjective cognitive decline serves as an early and potentially important indicator that individuals may be at risk for future cognitive decline or neurodegenerative conditions. However, there is a dearth of studies on factors influencing the experience of subjective cognitive decline in Black Americans, especially in Black American men.

**Objective:**

The current study explored correlates of subjective cognitive decline in Black American men.

**Participants:**

A total of 117 Black American men, with a mean age of 38.5 (SD = 7.14) years, participated in the study.

**Measurement:**

Participants completed a survey that assessed their demographic characteristics, self-rated health, neighborhood problems, length of residency in neighborhood, bodily symptoms, sleep comorbidities, sleep difficulties, and subjective cognitive decline. Linear regression analyses was performed and standardized beta coefficients were reported to describe the estimated independent effect of the predictor variables.

**Results:**

We found that socioecomic status (β = −.222, p=.003), bodily symptoms (β =.246, p=.005), length of residency in neighborhood (β =.157, p=.029), and sleep difficulties (β =.305, p<.001) were significant correlates of subjective cognitive decline among Black American men.

**Conclusion:**

These findings underscore the intricate roles of socioeconomic status, bodily symptoms, neighborhood factors, and sleep health in shaping subjective cognitive experiences in this population. Research on subjective cognitive decline can contribute to the early identification of individuals at risk for cognitive decline, allowing for timely interventions, lifestyle modifications, and potential preventive measures.

## Introduction

Dementia, particularly Alzheimer’s Disease (AD), is typically diagnosed when symptoms of cognitive decline have manifested and preventive protocols may no longer apply (1). Since late-stage dementia is irreversible, there has been increasing research interest in the preclinical stages, at which preventive measures may apply and/or progression may be delayed. In recent years, emphases have been placed on subjective cognitive decline (SCD) (2) as a precursor for cognitive decline to dementia (3–5). SCD —an individual’s self-reported experience of cognitive impairments or difficulties in memory and thinking abilities (3–6)—has been shown to be associated with cognitive decline. Liew (4) found that people with persistent SCD had a >75% probability of developing mild cognitive impairment and dementia within ten years. Jessen et al. (3) also reported that in a 12-year period, 82.7% of people with SCD developed dementia, while Robertson and Jacova (5) found SCD to be the most reliable predictor of dementia.

The overall prevalence of cognitive decline is higher in Americans racialized as Black compared to their White counterpart (7, 8). It is estimated that the prevalence of mild cognitive impairment is 32.6% among Black Americans, compared to White (20.3%) and Hispanic (26.8%) Americans (9). While research on SCD among Black Americans is evolving, existing studies suggest that this population may face a higher incidence of SCD compared to other racial groups (10–12). Gupta et al. (10) found SCD prevalence to be highest among Black Americans and linked to the prevalence of chronic conditions (e.g., heart attack, stroke) that are known precursors for cognitive decline to dementia. However, very little is known about the incidence of SCD in Black American men (hereafter referred to as Black men). Black men are often underrepresented in scientific studies, leading to a lack of comprehensive understanding of factors influencing their health outcomes (13). This underrepresentation hinders the ability to draw accurate conclusions and develop targeted interventions that address the unique challenges faced by Black men (13, 14).

Prior research links several factors to cognitive decline. Older age is consistently associated with a higher risk of cognitive decline, including conditions like Alzheimer’s disease and other forms of dementia (15). Educational attainment also plays a significant role, with higher levels of education often associated with a lower risk of cognitive decline and a reduced rate of cognitive decline over time (16). Furthermore, socioeconomic status, including factors such as income and occupation, can influence cognitive health, with individuals from lower socioeconomic backgrounds often experiencing higher rates of cognitive decline (17). These demographic factors may intersect to impact the risk of cognitive decline in Black men, and investigating them can help identify their complex interplay and inform interventions that address multiple levels of influence.

Studies point to associations between cognitive decline and sleep health (18, 19), particularly insomnia (18, 20) or difficulty initiating sleep (19). Neighborhood factors have also been linked to cognitive decline, including neighborhood problems (21, 22), neighborhood (dis) advantage (21, 22), neighborhood stress (23), access to green spaces (24), and pollution (25, 26). Understanding the role of these factors is crucial for addressing potential health disparities among Black men. However, the majority of the prior studies were on non-Black Americans or predominantly Black women. Thus, the current study explores the correlates of SCD among Black men to help inform community-level interventions and public health strategies for maintaining cognitive health and potentially reducing the risk of cognitive decline and AD.

## Methods

### Participants and Procedure

The study was designed as a cross-sectional study. Eligibility criteria for study participation included self-identifying as being a man (gender), Black American, 40+ years of age, able to read and understand English, and ability to provide consent for study participation. Data were collected via Amazon’s Mechanical Turk (MTURK) website—a crowdsourcing internet marketplace that allows community-based individuals to participate in academic research on Amazon’s platform for money (27). The published link was tailored to target Black men living in the United States. Attention checks were put in place to ensure that participants paid attention. In addition, we utilized location validation to ensure that only men in the United States completed the survey and the IP address blocker to prevent participants from submitting more than one response to the survey. Each online survey lasted approximately 45 minutes, and respondents received monetary compensation for study participation. The study was approved by the Institutional Review Board of Providence College, Rhode Island(#22-035).

### Measures

#### Subjective cognitive decline

The SCD measure (28) is a 21-item self-report questionnaire assessing memory changes, including everyday cognition and memory functioning. Sample questions are: “Do you think you have problems with your memory?” “Do you have difficulty remembering a conversation from a few days ago?” “How often is the following a problem for you: personal dates (e.g., birthdays),” and “How often is the following a problem for you: Phone numbers you use frequently?” Response options were dichotomous (yes = 1/no = 0) for 15 questions and Likert scale (i.e., always = 2, sometimes = 1, or never a problem = 0) for six questions. SCD score was computed for each individual by summing their score on the questions, with higher scores indicating greater reports of SCD. The mean score for this sample was 7.25 (SD = 5.48). The scale yielded a reliability score of Cronbach’s *α* = .87.

### Predictor variables

#### Neighborhood problems

The neighborhood problems were measured using a single-item question that assesses a participant’s perception of problems in their neighborhood. Participants were asked, “In your opinion, how will you rate the overall level of physical problems (e.g., litter, vandalism, noise, etc.) in your neighborhood.” The response options were on a 4-point Likert scale from 0 (no presence of physical problems) to 3 (high presence of physical problems). The mean score for this population was 1.30 (SD = 0.48), with higher scores indicating a greater perceived presence of neighborhood problems.

#### Length of residency in the current neighborhood

Participants were asked to self-indicate how long they have lived in their current neighborhood using a single question, “How long have you lived in your current neighborhood?”

#### Sleep difficulties

Sleep difficulties were assessed using the Pittsburgh Sleep Quality Index (PSQI) (29). PSQI is a 19-item questionnaire used to assess sleep quality and quantity. Most of the items are evaluated on a 4-point scale, and scores range from 0 to 21, with higher scores indicating poorer overall sleep quality. PSQI has seven component scores: sleep quality, sleep latency, sleep duration, sleep efficiency, sleep disturbance, sleep medication, and daytime sleep dysfunction. In this study, the sum of scores from the seven components reflects the presence or absence of sleep difficulties, with higher scores indicating a greater presence of sleep difficulties.

#### Sleep comorbidities

Participants were asked if they have the following: insomnia, snoring/sleep apnea, parasomnias, sleep paralysis, restless legs syndrome, periodic limb movement, circadian rhythms disorder, and narcolepsy. Their response option was Yes or No. If they answered yes, they were asked follow-up questions: “How long?” and “Were you diagnosed by a medical professional?” Sleep comorbidity scores were computed for each individual by summing their scores, with higher scores indicating a greater presence of sleep comorbidities.

#### Bodily symptoms

The participants were asked to reflect on their experience of bodily symptoms, including pain, aches, pressures in the body, breathing difficulties, and tiredness, and respond to 3 questions. The questions were, “Do your bodily symptoms stop you from working?” “Do your bodily symptoms stop you from concentrating on what you are doing?” and “Do your bodily symptoms stop you from enjoying yourself?” The response options were: Never (0), Seldom (1), Sometimes (2), Often (3), and Almost always (4). Scores range from 0–12, with a mean score of 3.03 (SD = 2.96). A bodily symptom score was computed for each individual by summing items, with higher scores indicating a higher presence of bodily symptoms that have been linked to sleep difficulties.

#### Self-rated health

A single-item measure of subjective health—“In general, how would you rate your current health status?”— was used to assess self-ratings of health. Response options were: “excellent (3),” “good (2),” “fair (1),” or “poor (0).”

#### Demographic characteristics

Four demographic variables were included in the analyses: age, marital status, education, and socioeconomic status. Socioeconomic status was assessed by asking participants to rate their social standing on a 10-rung ladder relative to others. Although the study was designed only to include Black American men, participants were asked to self-identify their race group (e.g., Black/African American, White/Caucasian American, Hispanic American, Asian American, etc.). It is our assumption that the participants who self-identify as Black American men are from various cultural backgrounds, such as Jamaican, Haitian, African, and Black (referring specifically to Black Americans). Those who did not self-identify as Black/African American were excluded from study participation. Age and education were included as continuous variables. However, marital status was also reported as a dichotomous variable (0=single/never married; 1=married).

### Data Analytic Strategy

Descriptive analyses were used to identify the sample’s demographic characteristics. A linear regression analysis was further specified to estimate the independent effect of age, education, marital status, socioeconomic status, neighborhood problems, bodily symptoms, self-rated health, length of residency, sleep difficulties, and sleep comorbidities on SCD. Standardized beta coefficients were reported to describe the estimated independent effect of the predictor variables. Statistical significance was determined with the probability of a Type I error, p ≤0.05. All statistical analyses were conducted using SPSS version 28.0 (SPSS Inc., Chicago, IL).

## Results

### The demographic characteristics of the sample

The distribution of the demographic and health characteristics for the total sample is shown in Table 1. The study included a sample of Black men (N=117), with a mean age of 47.50 (SD = 8.21) years. Approximately 55% reported being single. The average years of education of the total sample was 12.28 (SD = 2.50), and the majority (82.1%) reported having more than 12 years of education. Of the total sample, 79.5% self-rated their health as good or excellent, and 34.2% reported seeing 2–3 doctors, chiropractors, and other healers in the past year. Further demographic information can be found in Table 1.

**Table 1:** Sociodemographic characteristics of Black American men in the study of correlates of subjective cognitive decline (N=117)

**Variables**	**Mean (n)**	**SD (%)**
Age (years)	47.50	8.21
Education	12.38	2.50
>12 years	(96)	(82.1)
Marital status (single)	(64)	(54.7)
Socioeconomic status	5.06	1.56
Self-rated health	1.92	0.66
Excellent	(18)	(15.4)
Good	(75)	(64.1)
Fair	(21)	(17.9)
Poor	(3)	(2.6)
How often they see a doctor		
About once a month	(9)	(7.7)
About 4 times a year	(44)	(37.6)
Only very rarely	(53)	(45.3)
Almost never	(11)	(9.4)
How often they have been treated during the past year (For exam-ple, drugs, change of drugs, sur-gery, etc.).		
Always	(1)	(0.9)
Most of the time	(8)	(6.8)
About half of the time	(11)	(9.4)
Sometimes	(34)	(29.1)
Never	(63)	(53.8)
How many different doctors, chiropractors, and other healers they have seen in the past year.		
6 or more	(1)	(0.9)
4–5	(5)	(4.3)
2–3	(40)	(34.2)
1	(41)	(35.0)
0	(30)	(25.6)
Bodily symptoms	3.03	2.96
Neighborhood problems	1.30	0.48
Length of residency in neighbor-hood	8.14	3.59
Sleep difficulties	7.15	3.29
Sleep comorbidities	1.02	1.35

### Association between subjective cognitive decline and predictor variables

The independent associations of the study variables to SCD are shown in Table 2. Results showed that Black men with higher socioeconomic status were significantly more likely to report lower levels of SCD (β = −.222, p=.003). Black men who reported more bodily symptoms, such as pain or discomfort, were significantly more likely to report higher levels of SCD (β = .248, p=.005). Results further showed that a longer length of residency in a neighborhood was significantly associated with the likelihood of reporting higher levels of SCD (β = .157, p=.029). Finally, Black men who reported experiencing higher levels of sleep difficulties were significantly more likely to report higher levels of SCD (β = .305, p<.001). The linear regression model was significant (F (10,106) = 10.546, p<.001).

**Table 2:** Associations between subjective cognitive decline and predictor variables among Black American men (N=117)

				**95% CI**	
**Variables**	**β**	**SE**	**p-value**	**Lower**	**Upper**
Age	.045	.050	0.385	−.069	.130
Education	−.109	.173	0.171	−.582	.105
Marital status	.032	.840	0.676	−1.314	2.019
Socioecomic status	−.222	.255	0.003	−1.283	−.273
Self-rated health	−.150	.643	0.054	−2.525	.024
Bodily symptoms	.248	.161	0.005	.141	.779
Neighborhood problems	.129	.252	0.079	−.053	.945
Length of residency	.157	.108	0.029	.026	.453
Sleep difficulties	.305	.143	<.001	.225	.791
Sleep comorbidities	.125	.328	0.125	−.142	1.158
R^2^	.497				
df	10,106				
F	10.459				
p	<.001				

## Discussion

Past research suggests that SCD serves as an early and potentially important indicator that individuals may be at risk for future cognitive decline or neurodegenerative conditions. The current study investigated the correlates of SCD among Black men. We found that while higher levels of SES were associated with lower reports of SCD, greater presence of bodily symptoms, longer residency in a neighborhood, and greater presence of sleep difficulties were associated with higher reports of SCD among Black men.

Our finding that SES is negatively associated with SCD in Black men is in line with previous studies that found that lower SES is a risk factor for SCD (30, 31). We also found a significant relationship between bodily symptoms and SCD — increased bodily symptoms like pain, aches, pressures in the body, breathing difficulties, and tiredness increased the likelihood of Black men in the study reporting the presence of SCD. These results are somewhat supported by a recent prospective cohort study using eight waves of national data from the Health and Retirement Study (32). Du et al. (32) found that an increase in multimorbidity status, depressive symptoms, and instrumental activities of daily living limitations increased the risk of cognitive impairment in older adults. The Centers for Disease Control and Prevention posit that more adults and older adults with chronic disease report the presence of SCD compared to those without (33). Thus, bodily symptoms may be indicative of underlying health issues that could impact cognitive function. Further studies are recommended to examine if bodily symptoms are predictors of SCD in Black men when chronic diseases or presence of comorbidities, depression, anxiety, and functional disability are controlled for in the analysis.

The study revealed that the longer a person lived in a neighborhood, the greater the presence of SCD. Our findings suggest that the presence of SCD could be due to prolonged exposure to adversities such as environmental stressors, socioeconomic disadvantages, and social isolation. While neighborhood problems had a trending association with SCD in this study, other studies (21–22, 25, 26, 34) showed that living in disadvantaged neighborhoods may increase the risk of cognitive decline. People in these neighborhoods experience poor access to quality education, health care facilities, and public resources, high pollution (e.g., noise, air), and poor living conditions (35). Therefore, it is reasonable to expect that longer stays in neighborhoods with greater problems will increase the risk of cognitive decline. A geospatial analysis (e.g., using zip codes) may offer more information on the association between neighborhood problems, length of neighborhood residency, and cognitive decline among Black men.

Our study further revealed that a higher score for sleep difficulties was associated with higher SCD scores, indicating that Black men with sleep difficulties have an increased risk of SCD. This is in line with a study by Liu et al. (36), which found that self-reported shorter sleep duration and poor sleep efficiency are associated with dementia. Several underlying mechanisms have been proposed to explain these observations. Eide and colleagues (37) concluded that sleep deprivation impairs molecular clearance from the brain. Similarly, Holth et al. (38) found higher levels of AD biomarkers in sleep-deprived people. Bellesi (39) also found that sleep deprivation can lead to the death of nerve cells in the brain, which may cause or further worsen brain dysfunction. All these studies support our findings on sleep health and SCD in urban Black men.

Overall, our findings highlight the role of SES, sleep difficulties, length of residency in a neighborhood, and bodily symptoms in shaping subjective cognitive experiences among Black men. It illuminates the importance of considering a holistic approach when examining cognitive health, incorporating both individual and contextual determinants. This study’s emphasis on understanding these factors can contribute to the early identification of individuals at risk for cognitive decline, enabling timely interventions, lifestyle modifications, and potential preventive measures. It contributes valuable insights to the broader understanding of cognitive health disparities, emphasizing the need for nuanced and culturally sensitive approaches to address subjective cognitive decline in Black men.

Although this study demonstrated significant results, it has certain limitations. The current study was cross-sectional and did not assess changes in SCD over time. We did not capture a history of medical comorbidities, such as hypertension and diabetes, that are very common in Black Americans. Such information could offer additional biological insight into why some of the participants reported SCD. The data were collected via self-report, which has the potential for response bias from the participants, such as social desirability. Also, the participants were recruited from MTURK. The participant pool on MTurk may only partially represent the broader population, as it attracts more tech-savvy individuals who frequently participate in online tasks. Further, we did not include neuropsychological variables that are wellkn own precursors of cognitive decline, such as depression. Assessing such variables ensures a holistic understanding of cognitive health that can lead to more accurate diagnoses for men experiencing cognitive decline. Despite these limitations, our findings significantly contribute to the literature as they explore the trends of SCD and suggest the need for further studies to clarify factors associated with SCD in Black men.

## Conclusion

This study contributes valuable insights into the factors associated with SCD among Black men. The results reveal the relevance of SES, sleep difficulties, length of residency in a neighborhood, and bodily symptoms in cognitive well-being. Also, the study provides a nuanced perspective by identifying specific factors among Black men that may contribute to SCD. Given that SCD is a reported preindicator of neurological disorders such as Alzheimer’s disease, this research contributes to the early identification of individuals at risk for cognitive decline. The findings point to the need for the development of timely interventions, lifestyle modifications, and potential preventive measures tailored to the unique needs of Black men. Continued exploration of subjective cognitive decline within diverse populations is crucial for advancing our understanding of cognitive health disparities and promoting proactive measures for improved overall well-being.

## Data Availability

*Availability of data and material:* The current paper contains data that is part of a larger project. Details about the larger project are available from the first author. The study used standardized questionnaires that are accessible on Google Scholar.

## Notes

*Ethical approval:* The Institutional Review Board of Providence College, Rhode Island (#22-035).

## References

[1] Grueso S, Viejo-Sobera R. Machine learning methods for predicting progression from mild cognitive impairment to Alzheimer’s disease dementia: a systematic review. Alzheimers Res Ther. 2021;13:1–29. 10.1186/s13195-021-00900-w 34583745 10.1186/s13195-021-00900-w PMC8480074

[2] Si T, Xing G, Han Y. Subjective cognitive decline and related cognitive deficits. Front Neurol. 2020;11:493035. 10.3389/fneur.2020.00247 10.3389/fneur.2020.00247 PMC7248257 32508729

[3] Jessen F, Kleineidam L, Wolfsgruber S, Bickel H, Brettschneider C, Fuchs A, Kaduszkiewicz H, König HH, Mallon T, Mamone S, Pabst A. Prediction of dementia of Alzheimer type by different types of subjective cognitive decline. Alzheimers Dement. 2020; 16(12):1745–9. 10.1002/alz.12163 33140565 10.1002/alz.12163

[4] Liew TM. Trajectories of subjective cognitive decline, and the risk of mild cognitive impairment and dementia. Alzheimers Res Ther. 2020; 12:1–2. 10.1186/s13195-020-00699-y 10.1186/s13195-020-00699-y PMC7592368 33109275

[5] Earl Robertson F, Jacova C. A systematic review of subjective cognitive characteristics predictive of longitudinal outcomes in older adults. Gerontologist. 2023; 63(4):700–16. 35908232 10.1093/geront/gnac109

[6] Parfenov VA, Zakharov VV, Kabaeva AR, Vakhnina NV. Subjective cognitive decline as a predictor of future cognitive decline: a systematic review. Dement Neuropsychol. 2020;14:248–57. 32973979 10.1590/1980-57642020dn14-030007 PMC7500809

[7] Katz MJ, Lipton RB, Hall CB, Zimmerman ME, Sanders AE, Verghese J, Dickson DW, Derby CA. Age-specific and sex-specific prevalence and incidence of mild cognitive impairment, dementia, and Alzheimer dementia in blacks and whites: a report from the Einstein Aging Study. Alzheimer Dis Assoc Disord. 2012; 26(4):335–43. 10.1590/1980-57642020dn14-030007 22156756 10.1097/WAD.0b013e31823dbcfc PMC3334445

[8] Manly JJ, Jones RN, Langa KM, Ryan LH, Levine DA, McCammon R, Heeringa SG, Weir D. Estimating the prevalence of dementia and mild cognitive impairment in the US: the 2016 health and retirement study harmonized cognitive assessment protocol project. JAMA neurol. 2022; 79(12):1242–9. 10.1097/WAD.0b013e31823dbcfc 36279130 10.1001/jamaneurol.2022.3543 PMC9593315

[9] Rajan KB, Weuve J, Barnes LL, McAninch EA, Wilson RS, Evans DA. Population estimate of people with clinical Alzheimer’s disease and mild cognitive impairment in the United States (2020–2060). Alzheimers Dement. 2021; 17(12):1966–75. 10.1001/jamaneurol.2022.3543 34043283 10.1002/alz.12362 PMC9013315

[10] Gupta S. Racial and ethnic disparities in subjective cognitive decline: A closer look, United States, 2015–2018. BMC public health. 2021; 21:1–2. 10.1002/alz.12362 34162356 10.1186/s12889-021-11068-1 PMC8223389

[11] Jang Y, Haley WE, Choi EY, Franco Y. Racial/ethnic differences in correspondence between subjective cognitive ratings and cognitive impairment. Am J Geriatr Psychiatry. 2022; 30(5):627–35. 10.1186/s12889-021-11068-1 34862119 10.1016/j.jagp.2021.10.015

[12] Wooten KG. Racial and ethnic differences in subjective cognitive decline—United States, 2015–2020. MMWR. MMWR. 2023; 72. 10.1016/j.jagp.2021.10.015 10.15585/mmwr.mm7210a1 PMC10010752 36893045

[13] Esiaka DK, Wilson GB, Gluck MA. Strategies for Recruiting Older Black Men into Aging and Alzheimer’s Research. Prog Community Health Partnersh. 2024; 18(1):61–6. 10.15585/mmwr.mm7210a1 38661827

[14] Esiaka D, Yarborough CC, Fausto BA, Gluck MA. A mini-review of strategies for recruiting older African Americans to Alzheimer’s disease research. Community Health Equity Res Policy. 2022; 0272684X221118493. 10.1177/0272684X221118493 PMC11025449 36120808

[15] Alzheimer’s Association. 2024 Alzheimer’s Disease Facts and Figures. Alzheimers Dement. 2024: 20(5). 10.1002/alz.13809 PMC11095490 38689398

[16] Lenehan ME, Summers MJ, Saunders NL, Summers JJ, Vickers JC. Relationship between education and age-related cognitive decline: A review of recent research. Psychogeriatrics. 2015; 15(2):154–62. 25516261 10.1111/psyg.12083

[17] Lyu J, Burr JA. Socioeconomic status across the life course and cognitive function among older adults: An examination of the latency, pathways, and accumulation hypotheses. Journal of Aging and Health. 2016; 28(1):40–67. 26006338 10.1177/0898264315585504

[18] Cipriani G, Lucetti C, Danti S, Nuti A. Sleep disturbances and dementia. Psychogeriatrics. 2015;15(1):65–74. 10.1111/psyg.12069 25515641 10.1111/psyg.12069

[19] Zaheed AB, Chervin RD, Spira AP, Zahodne LB. Mental and physical health pathways linking insomnia symptoms to cognitive performance 14 years later. Sleep. 2023; 46(3):zsac262. 10.1093/sleep/zsac262 36309871 10.1093/sleep/zsac262 PMC9995792

[20] Sindi S, Kåreholt I, Johansson L, Skoog J, Sjöberg L, Wang HX, Johansson B, Fratiglioni L, Soininen H, Solomon A, Skoog I. Sleep disturbances and dementia risk: a multicenter study. Alzheimers Dement. 2018; 14(10):1235–42. 10.1016/j.jalz.2018.05.012 30030112 10.1016/j.jalz.2018.05.012

[21] Hunt JF, Vogt NM, Jonaitis EM, Buckingham WR, Koscik RL, Zuelsdorff M, Clark LR, Gleason CE, Yu M, Okonkwo O, Johnson SC. Association of neighborhood context, cognitive decline, and cortical change in an unimpaired cohort. Neurol. 202; 96(20):e2500–12. 10.1212/WNL.0000000000011918 10.1212/WNL.0000000000011918 PMC8205478 33853894

[22] Luo Y, Zhang L, Pan X. Neighborhood environments and cognitive decline among middle-aged and older people in China. J Gerontol B Psychol Sci Soc Sci. 2019; 74(7):e60–71. 10.1093/geronb/gbz016 30726959 10.1093/geronb/gbz016

[23] Choi E, Ailshire J. NEIGHBORHOOD STRESSORS AND EPIGENETIC AGING AMONG OLDER ADULTS IN THE US. Innov Aging. 2023; 7(Supplement_1):1088-. 10.1093/geroni/igad104.3495

[24] Chen X, Lee C, Huang H. Neighborhood built environment associated with cognition and dementia risk among older adults: a systematic literature review. SSM. 2022; 292:114560. 10.1016/j.socscimed.2021.114560 10.1016/j.socscimed.2021.114560 34776284

[25] Weuve J, D’Souza J, Beck T, Evans DA, Kaufman JD, Rajan KB, de Leon CF, Adar SD. Long-term community noise exposure in relation to dementia, cognition, and cognitive decline in older adults. Alzheimer Dement. 2021; 17(3):525–33. 10.1002/alz.12191 10.1002/alz.12191 PMC8720224 33084241

[26] Zhao YL, Qu Y, Ou YN, Zhang YR, Tan L, Yu JT. Environmental factors and risks of cognitive impairment and dementia: a systematic review and meta-analysis. Ageing Res Rev. 2021; 72:101504. 10.1016/j.arr.2021.101504 34755643 10.1016/j.arr.2021.101504

[27] Hauser DJ, Moss AJ, Rosenzweig C, Jaffe SN, Robinson J, Litman L. Evaluating CloudResearch’s Approved Group as a solution for problematic data quality on MTurk. Behav Res Methods. 2023; 55(8):3953–64. 10.3758/s13428-022-01999-x 36326997 10.3758/s13428-022-01999-x PMC10700412

[28] Gifford KA, Liu D, Romano RR, Jones RN, Jefferson AL. Development of a subjective cognitive decline questionnaire using item response theory: a pilot study. Alzheimer’s & Dementia: Diagnosis, Assessment & Disease Monitoring. 2015; 1(4):429–39. 10.1016/j.dadm.2015.09.004 10.1016/j.dadm.2015.09.004 PMC4750048 26878034

[29] Buysse DJ, Reynolds III CF, Monk TH, Berman SR, Kupfer DJ. The Pittsburgh Sleep Quality Index: a new instrument for psychiatric practice and research. Psychiatry res. 1989; 28(2):193–213. 2748771 10.1016/0165-1781(89)90047-4

[30] Künzi M, Joly-Burra E, Zuber S, Haas M, Tinello D, Da Silva Coelho C, Hering A, Ihle A, Laera G, Mikneviciute G, Stringhini S. The relationship between life course socioeconomic conditions and objective and subjective memory in older age. Brain Sciences. 2021; 11(1):61. 33418943 10.3390/brainsci11010061 PMC7825056

[31] Simons RL, Ong ML, Beach SR, Lei MK, Philibert R, Mielke MM. Direct and indirect effects of socioeconomic status and discrimination on subjective cognitive decline: A longitudinal study of African American women. J Gerontol B Psychol Sci Soc Sci. 2023; 78(5):799–808. 36810805 10.1093/geronb/gbad029 PMC10195880

[32] Du M, Tao L, Liu M, Liu J. Trajectories of health conditions and their associations with the risk of cognitive impairment among older adults: insights from a national prospective cohort study. BMC medicine. 2024; 22(1):20. 10.1186/s12916-024-03245-x 38195549 10.1186/s12916-024-03245-x PMC10777570

[33] Centers for Disease Control and Prevention. Chronic diseases and cognitive decline — a public health issue. 2023. https://www.cdc.gov/aging/publications/chronic-diseases-brief.html Accessed 18 Frbruary 2024.

[34] Delgado-Saborit JM, Guercio V, Gowers AM, Shaddick G, Fox NC, Love S. A critical review of the epidemiological evidence of effects of air pollution on dementia, cognitive function and cognitive decline in adult population. Science of the Total Environment. 2021; 757:143734. 10.1016/j.scitotenv.2020.143734 33340865 10.1016/j.scitotenv.2020.143734

[35] Steptoe A, Feldman PJ. Neighborhood problems as sources of chronic stress: development of a measure of neighborhood problems, and associations with socioeconomic status and health. Ann Behav Med. 2001; 23(3):177–85. 10.1207/s15324796abm2303_5 11495218 10.1207/S15324796ABM2303_5

[36] Liu R, Tang S, Wang Y, Dong Y, Hou T, Ren Y, Cong L, Liu K, Qin Y, Sindi S, Du Y. Self-reported sleep characteristics associated with dementia among rural-dwelling Chinese older adults: a population-based study. BMC neurol. 2022; 22:1–3. 34979998 10.1186/s12883-021-02521-0 PMC8722012

[37] Eide PK, Vinje V, Pripp AH, Mardal KA, Ringstad G. Sleep deprivation impairs molecular clearance from the human brain. Brain. 2021; 144(3):863–74. 10.1093/brain/awaa443 33829232 10.1093/brain/awaa443

[38] Holth JK, Fritschi SK, Wang C, Pedersen NP, Cirrito JR, Mahan TE, Finn MB, Manis M, Geerling JC, Fuller PM, Lucey BP. The sleep-wake cycle regulates brain interstitial fluid tau in mice and CSF tau in humans. Science. 2019; 363(6429):880–4. 10.1126/science.aav2546 30679382 10.1126/science.aav2546 PMC6410369

[39] Bellesi M. The effects of sleep loss on brain functioning. In Handbook of Behavioral Neuroscience. 2019; (30): pp. 545–556). Elsevier. 10.1016/B978-0-12-813743-7.00036-0

